# Food insecurity is a barrier to prevention of mother-to-child HIV transmission services in Zimbabwe: a cross-sectional study

**DOI:** 10.1186/s12889-015-1764-8

**Published:** 2015-04-25

**Authors:** Sandra I McCoy, Raluca Buzdugan, Angela Mushavi, Agnes Mahomva, Frances M Cowan, Nancy S Padian

**Affiliations:** University of California, 1950 Addison Avenue, Suite 202-8, Berkeley, CA 94704 USA; Ministry of Health and Child Welfare, Mkwati Building Corner 5th Street and Livingstone Avenue, Harare, Zimbabwe; Elizabeth Glaser Pediatric AIDS Foundation, 107 King George Road, Avondale, Harare, Zimbabwe; Centre for Sexual Health and HIV Research, 9 Monmouth Rd, Avondale West, Harare, Zimbabwe; University College London, London, UK

**Keywords:** Food security, Prevention of mother-to-child transmission of HIV, PMTCT cascade, HIV infection, Zimbabwe, Maternal health

## Abstract

**Background:**

Food insecurity (FI) is the lack of physical, social, and economic access to sufficient food for dietary needs and food preferences. We examined the association between FI and women’s uptake of services to prevent mother-to-child HIV transmission (MTCT) in Zimbabwe.

**Methods:**

We analyzed cross-sectional data collected in 2012 from women living in five of ten provinces. Eligible women were ≥16 years old, biological mothers of infants born 9–18 months before the interview, and were randomly selected using multi-stage cluster sampling. Women and infants were tested for HIV and interviewed about health service utilization during pregnancy, delivery, and post-partum. We assessed FI in the past four weeks using a subset of questions from the Household Food Insecurity Access Scale and classified women as living in food secure, moderately food insecure, or severely food insecure households.

**Results:**

The weighted population included 8,790 women. Completion of all key steps in the PMTCT cascade was reported by 49%, 45%, and 38% of women in food secure, moderately food insecure, and severely food insecure households, respectively (adjusted prevalence ratio (PR_a_) = 0.95, 95% confidence interval (CI): 0.90, 1.00 (moderate FI vs. food secure), PR_a_ = 0.86, 95% CI: 0.79, 0.94 (severe FI vs. food secure)). Food insecurity was not associated with maternal or infant receipt of ART/ARV prophylaxis. However, in the unadjusted analysis, among HIV-exposed infants, 13.3% of those born to women who reported severe household food insecurity were HIV-infected compared to 8.2% of infants whose mothers reported food secure households (PR = 1.62, 95% CI: 1.04, 2.52). After adjustment for covariates, this association was attenuated (PR_a_ = 1.42, 95% CI: 0.89, 2.26). There was no association between moderate food insecurity and MTCT in unadjusted or adjusted analyses (PR_a_ = 0.68, 95% CI: 0.43, 1.08).

**Conclusions:**

Among women with a recent birth, food insecurity is inversely associated with service utilization in the PMTCT cascade and severe household food insecurity may be positively associated with MTCT. These preliminary findings support the assessment of FI in antenatal care and integrated food and nutrition programs for pregnant women to improve maternal and child health.

## Background

It is widely recognized that attrition from the prevention of mother-to-child HIV transmission (PMTCT) cascade is a significant obstacle to achieving UNAIDS’ and the World Health Organization (WHO)’s goal to eliminate mother-to-child transmission by 2015 [1-4]. The PMTCT cascade is a series of services that HIV-positive pregnant women and their infants need to receive in order to prevent HIV transmission, including antenatal care (ANC), HIV testing, and antiretroviral therapy (ART) or antiretroviral (ARV) prophylaxis [5]. However, 49% of HIV-infected pregnant women in sub-Saharan Africa are lost between ANC registration and delivery and miss some or all essential PMTCT services [6]. Furthermore, high rates of loss to follow-up among women initiating ART under ‘Option B+’, [7] WHO’s PMTCT strategy whereby all pregnant and breastfeeding women receive lifelong ART, [8] has renewed emphasis on the importance of reducing barriers to uptake of PMTCT services.

In many settings, economic factors are cited as barriers to ANC and PMTCT services, [9,10] including facility-based delivery, [11] initiation of ART and ARV prophylaxis, and retention in HIV care [12]. Here we focus on one aspect of socioeconomic position: food security. People are considered food secure when they have adequate physical, social, and economic access to sufficient, safe and nutritious food that meets their dietary needs and food preferences for an active and healthy life [13]. Food insecurity is increasingly recognized as exacerbating the HIV/AIDS epidemic by increasing engagement in HIV-related risk behaviors, [14-17] and among people living with HIV, undermining ART adherence and retention in care [18-20]. However, few studies have examined the relationship between food insecurity and utilization of PMTCT services and MTCT [19,20].

There are several pathways through which food insecurity might affect women’s use of health services and increase vertical HIV transmission [21]. First, food insecurity might result in avoidance or delay of maternal health services because of its overlap with socioeconomic position [22,23] and the real or perceived costs of ANC, facility delivery, and/or HIV prevention and care services. Second, food insecurity is associated with undernutrition, which among HIV-infected women is associated with preterm delivery, low birth weight, and MTCT [24-26]. In addition, food insecure HIV-infected women may be less likely to adhere to ART/ARV prophylaxis, [27] and they may exclusively breastfeed their infants for shorter periods of time, heightening the risk of onward transmission as they resort to mixed feeding [28,29]. Lastly, food insecurity may influence women’s receipt of PMTCT services through mental health pathways related to the anxiety and stress associated with real or perceived hunger [30,31]. Stress and depression, in turn, may affect service utilization and obstetric and child health outcomes [32,33]. Together, these compelling pathways support the hypothesis that food insecurity may undermine global efforts to achieve elimination of mother-to-child HIV transmission.

We examined the relationship between food security and PMTCT in Zimbabwe, where a generalized HIV epidemic coexists with food insecurity, hunger, and undernutrition. Thirty-three percent of Zimbabwe’s population is undernourished, [34] and the 2013 Global Hunger Index was 16.5, indicating serious levels of hunger [35]. Moreover, 12% of pregnant women are HIV-positive [36]. The objectives of our analyses were to: 1) determine the prevalence of food insecurity among women with a recent birth; 2) explore whether food insecurity is associated with receipt of PMTCT-related services; and 3) examine the association between food insecurity and MTCT.

## Methods

### Study population

We analyzed data from a 2012 cross-sectional survey of mother/caregiver-infant pairs conducted as part of the impact evaluation of Zimbabwe’s Accelerated National PMTCT Program [5,37]. The survey targeted women who were ≥16 years old and biological mothers or caregivers of infants (alive or deceased) born 9–18 months earlier in order to capture MTCT during pregnancy, delivery and breastfeeding. The primary outcomes of the impact evaluation were MTCT and HIV-free infant survival. Because the analyses presented in this paper use data from the 2012 survey, which were the baseline data for the parent impact evaluation, we describe the association between food security and engagement in PMTCT services *before* the Ministry of Health and Child Care’s (MoHCC) implementation of PMTCT strategy ‘Option A’. We restricted the sample of 9,018 mothers and caregivers to 8,662 biological mothers and their eligible infants by excluding 356 (3.9%) caregiver/infant pairs.

### Sampling strategy and data collection

The two-stage sampling strategy has been previously described [37,38]. Five provinces (Harare, Mashonaland West, Mashonaland Central, Manicaland, and Matabeleland South) were purposefully selected to include Zimbabwe’s capital, rural communities with higher and lower HIV prevalence, and both Shona and Ndebele ethnic groups. In the first stage, we randomly selected 157 catchment areas from 699 health facilities offering PMTCT services, proportionate to the number of facilities per district. In the second stage, in each catchment area, a pre-determined proportion of eligible infants was randomly sampled, depending on the size of the catchment area. Potentially eligible infants and their mothers/caregivers were identified by pooling information from: 1) community health workers, 2) immunization registers from both sampled and nearby health facilities, and 3) peer referral. Together, this approach efficiently identified eligible participants without screening all households and captured mother-infant pairs who did not utilize any health services and those who accessed care outside of their area of residence. Mothers providing written informed consent completed an anonymous interviewer-administered survey about maternal and household demographics, health services accessed during pregnancy and after delivery, and behaviors germane to MTCT (e.g., breastfeeding).

### Food security status

Household food security was determined with a subset of questions from the Household Food Insecurity Access Scale (HFIAS) [30]. Due to interview time constraints, we selected three questions for inclusion, one from each domain of food access of the HFIAS: 1) anxiety and uncertainty about household food supply; 2) insufficient quality, including food variety and preferences; and 3) insufficient food intake and its physical consequences [30,39]. Women were asked how often, in the last 4 weeks, they worried that their household would not have enough food (anxiety/uncertainty), how often they were not able to eat preferred foods because of lack of resources (insufficient quality), and whether anyone in the household went to bed hungry (insufficient intake).

Based on the distribution of these responses, consideration of the recommendations for categorizing responses to the full HFIAS, and examination of other food security scales [40], we determined an algorithm to classify households into three mutually exclusive groups: food secure, moderately food insecure, and severely food insecure. Severe food insecurity was defined as ≥1 household member going to bed hungry (even if infrequently or rarely) or “often” worrying (more than 10 times in the last month) about food access or food quality. Households were classified as having moderate food insecurity if they “sometimes” (3–10 times in the last month) worried about food access or quality. Food secure households experienced either none of the food insecurity conditions or they only rarely worried about food access or quality. We assumed that household food security status in the previous 4 weeks was strongly correlated with what food security status would have been during pregnancy, 9–18 months prior. We excluded seven women without food security information from the analysis.

### Assessment of HIV status

Living mothers and infants provided blood spot samples for HIV testing, which were air-dried onto filter papers and stored at room temperature until biweekly transport to the laboratory. Maternal samples were tested for HIV-1 antibody using AniLabsytems EIA kit (AniLabsystems Ltd, OyToilette 3, FIN-01720, Vantaa, Finland) with positive specimens confirmed using Enzygnost Anti-HIV 1/2 Plus ELISA (Dade Behring, Marburg, Germany) and discrepant results resolved by Western Blot. Samples from HIV-exposed infants and infants of mothers with unavailable samples were tested for HIV with DNA polymerase chain reaction (Roche Amplicor HIV-1 DNA Test, version 1.5). Results were available for 97.8% and 97.2% of women and HIV-exposed infants, respectively. Women were able to receive their HIV test results at the local health facility up to 3 months after the survey using a card with a barcode of their unique identification number.

### Data analyses

We first compared socio-demographic characteristics and service utilization stratified by food security status. We examined the following maternal health services: ANC (any and the WHO-recommended ≥4 visits [41]), gestational age in weeks at ANC registration (WHO recommends the first visit should occur in first trimester [41]), HIV testing during ANC or labor and delivery (or prior knowledge of HIV-positive serostatus), facility-based delivery, and postnatal visit attendance (6–8 weeks postpartum). Among HIV-infected women, we examined reported use of maternal and infant ART/ARV prophylaxis, infant co-trimoxazole prophylaxis, exclusive breastfeeding (≥1 month), and MTCT, stratified by food security status. We also examined a combined category indicating “completion” of the cascade including the following key services: ≥4 ANC visits, HIV testing, facility-based delivery, postnatal visit attendance, and among HIV-infected women, report of maternal and infant ART or ARV prophylaxis and co-trimoxazole prophylaxis. Missing values of PMTCT services were <1%; in those few cases, women were classified as not having received the service.

We conducted an exploratory analysis to describe the association between food security and completion of the PMTCT cascade and MTCT using Poisson regression models. With cross-sectional data, the exponentiated parameter estimates represent prevalence ratios (PR) [42-44]. The fully adjusted models contain all covariates specified *a priori* for inclusion (see below) and key services or behaviors not hypothesized to lie on the causal pathway between food insecurity and the outcome. Covariates with variance inflation factors >10 (indicating multicollinearity) were examined for correlation with food security status and if necessary, excluded [45]. We present PRs and 95% confidence intervals (CI) computed with linearized standard errors to account for the sample design.

Several covariates, which likely preceded pregnancy, were considered for inclusion in models as potential confounders: province, mother’s age, religion, tribe, being married or having a regular sexual partner, mother’s highest educational level, household size, lifetime births, and the building materials of the best building on the homestead. Additionally, we created a household asset index, divided into quartiles, using principal component analysis with a polychoric correlation matrix [46-48]. We also included a variable to indicate the infant’s age in months at the time of the survey (or age the infant would have been, if deceased), indicative of the time elapsed between the pregnancy and the interview to account for recall bias. No more than 1% of any covariate was missing. All analyses were conducted with STATA 12 (College Station, Texas) and were weighted to account for the varying sampling fraction by catchment area and 1.1% survey non-response.

### Human subjects protection

The Medical Research Council of Zimbabwe and the ethical review boards at the University of California, Berkeley and University College London approved this study.

## Results

### Participant characteristics

The weighted population included 8,790 eligible mothers (based on 8,655 observations). The average age of women was 26.7 years, 93% were married or had a regular sexual partner, and they had an average of 2.7 lifetime births (Table 1). Overall, 4,305 (49%) women reported living in food secure households, 2,906 (33%) were living in moderately food insecure households, and 1,578 (18%) were living in severely food insecure households. Women living in moderately or severely food insecure households were less likely to be from Harare, less likely to have a husband or main partner (92% vs. 94%), had less education, fewer assets, and larger household sizes (5.3 vs. 5.0 members). Of the women with HIV test results, 1,075 (12.4%) were HIV-infected; including 9.8%, 12.4%, and 19.4% of women living in food secure, moderately food insecure, and severely food insecure households, respectively (p < 0.01).

**Table 1:** **Sociodemographic characteristics of participants, Zimbabwe, 2012; Women were ≥16 years old and mothers of infants (alive or deceased) born 9–18 months prior to the interview** ^**a**^

**Characteristic**		**Total (N = 8,790)**	**Household food security status** ^**b**^		
			**Food secure (n = 4,305)**	**Moderate food insecurity (n = 2,906)**	**Severe food insecurity (n = 1,578)**
		**N (%)**	**N (%)**	**N (%)**	**N (%)**
Province					
	Harare	1,529 (17.4)	930 (21.6)	439 (15.1)	160 (10.1)
	Manicaland	3,564 (40.6)	1,449 (33.7)	1,366 (47.0)	749 (47.5)
	Mashonaland Central	1,503 (17.1)	818 (19.0)	504 (17.3)	181 (11.5)
	Mashonaland West	1,339 (15.2)	713 (16.6)	413 (14.2)	214 (13.6)
	Matabeleland South	855 (9.7)	396 (9.2)	184 (6.3)	275 (17.4)
Age, years (mean, SE)		26.7 (0.09)	26.2 (0.12)	27.0 (0.12)	27.4 (0.21)
Married or has a regular sexual partner		8,152 (92.7)	4,028 (93.6)	2,691 (92.6)	1,432 (90.8)
Education, highest completed					
	No education	274 (3.1)	99 (2.3)	123 (4.2)	52 (3.3)
	Primary school (Standard 7)	2,458 (28.0)	966 (22.4)	854 (29.4)	638 (40.4)
	Some secondary school	2,469 (28.1)	1,120 (26.0)	855 (29.4)	494 (31.3)
	“O” Level or more (Grade 11)	3,589 (40.8)	2,120 (49.3)	1,073 (36.9)	396 (25.1)
Ethnicity					
	Shona	7,341 (83.5)	3,646 (84.7)	2,516 (86.6)	1,179 (74.7)
	Ndebele	586 (6.7)	297 (6.9)	116 (4.0)	172 (10.9)
	Kalanga/Other	862 (9.8)	361 (8.4)	274 (9.4)	227 (14.4)
Household size (mean, SE)		5.2 (0.05)	5.0 (0.06)	5.2 (0.05)	5.5 (0.08)
Asset Index (quartile)					
	1st (lowest)	2,463 (28.0)	862 (20.0)	874 (30.1)	726 (46.0)
	2nd	1,624 (18.5)	748 (17.4)	559 (19.2)	317 (20.1)
	3rd	2,022 (23.0)	1,016 (23.6)	687 (23.6)	320 (20.3)
	4th (highest)	2,681 (30.5)	1,679 (39.0)	786 (27.1)	216 (13.7)
Lifetime births (mean, SE)		2.7 (0.04)	2.4 (0.04)	2.8 (0.04)	3.1 (0.06)
Infant alive		8,726 (99.3)	4,278 (99.4)	2,882 (99.2)	1,565 (99.2)
Infant’s age, months (mean, SE)^c^		13.7 (0.04)	13.6 (0.05)	13.7 (0.07)	13.6 (0.08)
Mother HIV-infected		1,075 (12.4)	414 (9.8)	358 (12.4)	304 (19.4)

SE: Linearized standard error.

^a^Weighted counts and proportions presented in the table. Numbers may not sum to column totals due to missing data. Percentages may not add to 100 due to rounding.

^b^Food security determined from a subset of questions from the Household Food Insecurity Access Scale (HFIAS) [30].

^c^Age of infants who were alive as well as the age deceased infants would have been at the time of the survey.

### Food insecurity and receipt of maternal health services

Food insecurity was inversely associated with use of ANC services, with 95%, 94%, and 92% of women from food secure, moderately food insecure, and severely food insecure households reporting attendance at ≥1 ANC visit, respectively (p < 0.01, Table 2), although food insecurity was not associated with ≥4 ANC visits nor the timing of the first ANC visit. Compared to women from food secure households, women from moderately or severely food insecure households were significantly less likely to know their HIV status during pregnancy or labor and delivery, were significantly less likely to deliver in a health facility, and were less likely to report attending the postnatal visit. Overall, completion of all key steps in the PMTCT cascade was reported by 49% of women from food secure households, 45% of women from moderately food insecure households, and 38% of women from severely food insecure households (adjusted PR_a_ = 0.95, 95% CI: 0.90-1.00 (moderate food insecurity vs. food secure), PR_a_ = 0.86, 95% CI: 0.79, 0.94 (severe food insecurity vs. food secure), adjusted Wald test p < 0.01, Table 3). There was no association between food insecurity and completion of the cascade when the analysis was restricted to HIV-infected women.

**Table 2:** **Food security status and receipt of services in the PMTCT cascade, Zimbabwe, 2012** ^**a**^

**Service**		**Total (N = 8,790)**	**Household food security status** ^**b**^		
			**Food secure (n = 4,305)**	**Moderate food insecurity (n = 2,906)**	**Severe food insecurity (n = 1,578)**
		**N (%)**	**N (%)**	**N (%)**	**N (%)**
Antenatal care (ANC)					
	Any	8,287 (94.2)	4,091 (95.0)	2,737 (94.3)	1,450 (92.0)**
	≥4 visits	5,627 (64.5)	2,801 (65.6)	1,855 (64.3)	971 (62.2)
	Gestational age (months) at booking (mean, SE)	5.1 (0.06)	5.1 (0.08)	5.1 (0.06)	5.0 (0.08)
Tested for HIV infection in ANC or labor and delivery (L&D) or knew HIV-infected		8,117 (92.4)	4,043 (93.9)	2,689 (92.5)	1,385 (87.8)**
Health facility delivery		6,747 (76.8)	3,469 (80.6)	2,199 (75.7)	1,079 (68.4)**
Postnatal visit attendance		8,109 (92.4)	4,040 (93.9)	2,647 (91.3)	1,422 (90.1)**
If HIV-infected (n = 1,075):					
	Received ART or ARVs	639 (59.5)	242 (58.6)	215 (60.2)	182 (59.9)
	Infant prophylaxis	673 (62.9)	250 (61.1)	227 (63.4)	196 (64.7)
	Co-trimoxazole prophylaxis	475 (44.2)	184 (44.6)	166 (46.5)	124 (40.9)
	Exclusive breastfeeding (ever)	959 (95.3)	364 (95.9)	314 (92.0)	281 (98.3)**
	Infant infected	93 (9.0)	33 (8.2)	22 (6.2)	39 (13.3)**
**Received all key maternal health services** ^**c**^		4,027 (45.8)	2,121 (49.3)	1,301 (44.8)	605 (38.3)**
	HIV-infected women	299 (27.8)	111 (26.7)	107 (30.0)	82 (26.8)
	HIV-uninfected women	3,727 (49.1)	2,011 (52.8)	1,194 (47.4)	523 (41.5)**

SE: Linearized standard error.

Design-based chi-squared p-value: *p < 0.05, **p < 0.01.

^a^Weighted counts and proportions presented in the table. Numbers may not sum to column totals due to missing data. Percentages may not add to 100 due to rounding.

^b^Food security determined from the responses to a subset of questions from the Household Food Insecurity Access Scale (HFIAS) [30].

^c^Completed at least 4 ANC visits, was tested for HIV infection or already knew HIV-positive serostatus, delivered infant in a health facility, and attended the postnatal visit. Among HIV-infected women, must also have reported maternal and infant ART or ARV prophylaxis and receipt of infant co-trimoxazole prophylaxis.

**Table 3:** **Association between household food security and completion of services in the PMTCT cascade and MTCT, Zimbabwe, 2012**

**Household food security status**	**All women: completion of key PMTCT services** ^**a**^				**HIV-infected women: MTCT** ^**b**^			
	**Unadjusted**		**Adjusted**		**Unadjusted**		**Adjusted**	
	**PR**	**95% CI**	**PR**	**95% CI**	**PR**	**95% CI**	**PR**	**95% CI**
Food secure	1	---	1	---	1	---	1	---
Moderate food insecurity	0.91	(0.86, 0.96)**	0.95	(0.90, 1.01)	0.75	(0.46, 1.24)	0.68	(0.43, 1.08)
Severe food insecurity	0.78	(0.70, 0.86)**	0.86	(0.79, 0.94)**	1.62	(1.04, 2.52)*	1.42	(0.89, 2.26)

PR: prevalence ratio; CI: confidence interval.

P-value: *p < 0.05, **p < 0.01.

^a^Regression model of a weighted sample of 8,655 women. Outcome: combined variable indicating at least 4 ANC visits, tested for HIV infection or already knew HIV-positive serostatus, delivered infant in a health facility, attended the postnatal visit, and among HIV-infected women, report of maternal and infant ART or ARV prophylaxis and receipt of infant co-trimoxazole prophylaxis. Adjusted model includes province, maternal age, whether the woman has a husband or regular partner, education, tribe, religion, household size, building material of the best structure, an asset index created using principal component analysis, and whether the infant was alive at the time of the survey.

^b^Regression model of a weighted sample of 1,058 HIV-infected women who had infants with HIV test results. Adjusted model includes province, maternal age, whether the woman has a husband or regular partner, education, tribe, religion, household size, building material of the best structure, an asset index created using principal component analysis, and whether the infant was alive at the time of the survey.

### Food insecurity and MTCT

Food insecurity was not associated with maternal or infant receipt of ART/ARV prophylaxis. However, women living in severely food insecure households were the most likely to have ever exclusively breastfed their infant (98%, vs. 96% of women in food secure and 92% of women in moderately food insecure households, p < 0.01). Of HIV-exposed infants, 13.3% of infants from severely food insecure households were HIV-infected compared to 8.2% of infants from food secure households (PR = 1.62, 95% CI: 1.04, 2.52, Tables 2 & 3). After adjustment for covariates, this association was attenuated (PR_a_ = 1.42, 95% CI: 0.89, 2.26). There was no increased likelihood of MTCT among women from moderately food insecure households in unadjusted or adjusted analyses (PR_a_ = 0.68, 95% CI: 0.43, 1.08).

## Discussion

In this analysis of women with a recent birth in Zimbabwe, we found that more than half reported living in moderate or severely food insecure households in the month prior to the survey. Compared to women from food secure households, women from food insecure households were more likely to be HIV-infected. Consistent with previous qualitative studies, [49] we found that food insecurity may be an important barrier to uptake of some PMTCT services: in unadjusted analyses, food insecurity was inversely associated with ANC, knowing one’s HIV status, facility-based delivery, and postnatal visit attendance. When services were examined together, and after adjustment for covariates, women who reported severe food insecurity were 14% less likely to complete all recommended maternal and infant health services for PMTCT compared to food secure women. Although the effect sizes are modest and absolute differences are small, these findings suggest that among a subgroup of pregnant women, severe food insecurity is an important barrier to some maternal health services.

Among HIV-infected women, we unexpectedly found that there was no association between food insecurity and completion of the PMTCT cascade. This might be due to the small sample size, women’s motivation to protect their infant from HIV infection, or nutritional support provided during pregnancy and postpartum that partially mitigates household food insecurity. However, women from severely food insecure households were 42% more likely to have an HIV-infected infant compared to women from food secure households, although this finding was not statistically significant after adjustment for covariates. Although food security’s association with vertical transmission has been speculated, [21,50] this analysis is the first, to our knowledge, to provide empirical data supporting this hypothesis. We must nevertheless interpret these findings with caution because a strong ‘dose-response’ relationship between food insecurity and MTCT was not observed, as women in moderately food insecure households had the lowest proportions of exclusive breastfeeding and MTCT.

Although we found that food insecurity was associated with ever attending ANC, we found no association between food insecurity and attending the WHO-recommended ≥4 ANC visits or the timing of ANC registration. There are several potential explanations for this finding. In facilities that charge a fee, the fees often cover the bundle of services from ANC booking through the 6-week postnatal visit. Thus, once engaged in ANC, women may be retained in the cascade and continue to receive ANC care and deliver at the health facility. Women who don’t attend ANC may therefore also be more likely to deliver at home and miss the postnatal visit. Although this study found only small but statistically significant reductions in utilization of each service among women who reported being moderately or severely food insecure, losses from the cascade are additive, as was demonstrated by a cohort study in Cameroon, Côte d’Ivoire, South Africa, and Zambia, where unremarkable levels of attrition of HIV-infected mothers from individual steps in the cascade coupled with poor adherence resulted in 49% of HIV-exposed infants not being protected by a prophylactic ARV regimen [51].

An unanswered question is the pathway(s) through which food insecurity might impede service utilization. It is possible that food insecurity in this analysis is simply a proxy for poverty, and our findings are reflective of the difficult choices food insecure women must make between food (and other goods and services) and the costs associated with health care, including transport and fees. Certainly, food insecurity is highly correlated with socioeconomic position [22,23] and has been shown to be the strongest measure of socio-economic position associated with HIV and HSV-2 risk among young women in Zimbabwe, potentially due to engagement in risk behavior to obtain food or other essential goods and services [52]. This might be the most likely explanation for the inverse association we identified between food insecurity and ANC and other services that require or are perceived to require payment. Although Zimbabwe is moving toward elimination of user fees for maternal and child health services, some facilities still charge a fee that may be cost-prohibitive.

However, this economic explanation does not fully explain the finding that severe food insecurity was not associated with receipt of maternal and infant ART/ARVs, but may nevertheless be associated with MTCT, although the width of the confidence limits suggests substantial uncertainty in this association. One explanation might be that although both food secure and insecure women and infants were equally likely to receive ART/ARV prophylaxis, women who were food insecure were less likely to adhere to treatment. Food insecurity is known to reduce ART adherence in non-pregnant populations, [27] although this study is not able to test this hypothesis. Another possible explanation is more complex: severe food insecurity increases MTCT risk due to cumulative loss of women from the cascade coupled with an increased risk of MTCT associated with undernutrition [25,26] and the possible increased propensity for mixed feeding [28]. This study was unable to explore more complex patterns of breastfeeding (such as duration of exclusive breastfeeding) to support or refute this hypothesis, and our simple measure of exclusive breastfeeding (ever) found that nearly all women exclusively breastfed at some point. Furthermore, this secondary data analysis used cross-sectional data from a study that was not designed to specifically examine this question, so we do not have prospective data nor information on several causal intermediates, including nutritional indicators such as women’s body mass index (BMI) and micronutrient status, to examine these pathways. Nonetheless, these data provide both compelling empirical evidence about these hypothesized relationships and also reveal important gaps for future research.

An important issue when considering these findings is the measurement of food security [53,54]. In this study, household food security was measured in the 4 weeks prior to the survey, a common reference period used by other scales [30,55,56] in order to balance the tradeoffs between recall bias (which favors a shorter recall period) and ‘telescoping errors’ (a phenomenon associated with short recall periods whereby events outside of the recall window are erroneously reported) [57,58]. We made the essential temporality assumption that a household’s recent food security status was highly correlated with its food security status during the woman’s pregnancy, 9–18 months prior. The validity of this assumption is unknown; it depends on how much household food access changed over time and season, which was not measured in the survey. Nevertheless, this issue could be overcome in future prospective studies by conducting a simple food security assessment at ANC registration to identify food insecure women who are at risk of both undernutrition and of missing key PMTCT services.

Another key measurement issue is that food security was measured using only part of the HFIAS, a validated scale, which adds some uncertainty to the classification of food security. Nevertheless, our inclusion of a question from each dimension of food access [30] in addition to the correlation between our parameterization of food security and other dimensions of household socio-economic position increases confidence in our classification scheme. A strength of our measurement of food security is that women themselves reported household food security status, the individuals who are typically responsible for a household’s food supply and meal preparation in Zimbabwe. This is also important because food insecurity at the individual level may be prevalent even in wealthier households due to unequal intra-household allocation of food, which, for example, can result in women eating last or having less access to fats, protein, or micronutrient-rich foods [59-61]. Nevertheless, as with all measures of food insecurity, food security may be subject to underreporting because of its sensitive nature [62].

Our analysis has other important limitations. We used cross-sectional data and therefore cannot make inferences about causation. Further, women self-reported receipt of healthcare services. Moreover, although our data are representative of the communities from which the sample was selected, they are not representative of all regions in Zimbabwe, and it is possible that the relationship between food insecurity and service utilization are different in other parts of the country. In addition, although our strategy to create a sampling frame of 9–18 month old infants in the community was comprehensive, it is possible that some mother-infant pairs were missed. Lastly, women’s and infant’s HIV status was measured at the time of the survey, 9–18 months postpartum. Although we have assumed that women who were HIV-infected at the time of the survey were also HIV-infected during their pregnancy, it is possible that a small proportion were infected during pregnancy or postpartum. Likewise, infants who were still breastfeeding at the time of the survey remained at risk of MTCT, so we may not have captured all possible infant infections.

## Conclusions

This analysis is the first to our knowledge to examine the association between food insecurity and receipt of services in the PMTCT cascade. Our findings suggest that severe food insecurity may impede the receipt of some services among pregnant and postpartum women and may influence MTCT; these relationships will need to be confirmed by other prospective observational studies. In addition, the growing body of studies that examine the effect of food and cash transfers as a mechanism to mitigate food insecurity and improve HIV-related outcomes [63,64] will also contribute to understanding these relationships, including whether food assistance programs have unexpected spillover benefits on maternal and child health. Regardless of its effect on MTCT, the high prevalence of food insecurity among women with a recent birth in Zimbabwe provides additional support for integrated food and nutrition programs for pregnant women.

## Abbreviations

ANC: Antenatal care
ARV: Antiretroviral
ART: Antiretroviral therapy
CI: Confidence interval
HFIAS: Household Food Insecurity Access Scale
MoHCC: Ministry of Health and Child Care
MTCT: Mother-to-child HIV transmission
PR: Prevalence ratio
PMTCT: Prevention of mother-to-child HIV transmission
WHO: World Health Organization
